# Integrated care for the management of ageing-related non-communicable diseases: current gaps and future directions

**DOI:** 10.1007/s40520-020-01533-z

**Published:** 2020-04-10

**Authors:** Alessandro Monaco, Katie Palmer, Alessandra Marengoni, Stefania Maggi, Tarek A. Hassan, Shaantanu Donde

**Affiliations:** 1 HEC, Paris, France; 2 Oliba, Via Federico Cesi 30, 00193 Rome, Italy; 3 Department of Clinical and Experimental Sciences, University of Brescia, Brescia, Italy; 4 Aging Branch, CNR-Neuroscience Institute, Padua, Italy; 5 Upjohn (Division of Pfizer), New York, NY USA; 6 Upjohn (Division of Pfizer), Surrey, UK

**Keywords:** Chronic disease, Ageing, NCD, Clinical practice, Successful ageing, Integrated care

## Abstract

Due to the increase in the older population in Europe and associated rise in the absolute number of persons with Non-Communicable Diseases (NCDs), it is becoming increasingly important to find ways to promote healthy ageing, which is defined as the process of developing and maintaining the functional ability that enables well-being in older age. Older persons with NCDs can have complex care needs due to the increased risk of frailty, multimorbidity, and polypharmacy. However, current health systems in Europe often provide fragmented care for older people with NCDs; many receive disjointed care from numerous specialists or via different levels of care. In the current article, we discuss barriers and challenges in implementing integrated care models in European settings for older NCD patients. Specifically, we discuss the need for greater use of case managers in the care and treatment persons with complex care needs as well as the lack of training and education in healthcare professionals on topics related to multimorbidity, frailty, and polypharmacy. We discuss the limitations that arise from the current focus on disease-specific guidelines and care models that do not take comorbid conditions into account, and the lack of good quality evidence that evaluates the effectiveness of integrated care interventions, especially in European health settings. We highlight the importance of evaluating and monitoring mental health in conjunction with somatic symptoms in NCD patients and discuss the integral role of information and communication technology in healthcare to streamline integrated care processes and help to achieve better outcomes for patients.

## Population ageing in Europe and non-communicable disease (NCDs)

In Europe the number of older persons is rapidly increasing; in 2014 older persons accounted for 18.5% of the total population yet it is estimated that this will increase to 28.7% by 2080 [1]. Further, the number of very old people (aged 80+) is projected to more than double from 2014 to 2080 [1]. In conjunction with this population ageing, it is expected that there will be an increase in absolute numbers of non-communicable diseases (NCD), especially those that are age-related such as dementia disorders as well as cardio- and cerebrovascular diseases. NCDs are often associated with frailty [2–7] leading to numerous negative outcomes including unplanned hospitalization, disability, decreased quality of life, polypharmacy, and mortality [8–10]. In addition, as a person ages, there is an increased risk of having more than one comorbid condition, which is usually referred to as “multimorbidity”. The majority of persons aged 60 and over have multimorbidity [11]. Many people with NCDs, especially multimorbid patients, have complex care needs and are more likely to have complex drug regimens and polypharmacy and related care issues such as low adherence to care and treatment plans and adverse drug reactions [5, 12, 13].

Regardless of the disruptive effect of these global trends, institutions are finding it challenging to identify and implement solutions to intercept the evolving social and health needs of the population; technologies are ubiquitous and accessible yet are far from being efficiently integrated into the care process. Education as well as dynamics in the acceptance of innovation may be part of the issue but there is clearly a multi-systemic resistance to change that is preventing the health ecosystem to adopt modern and cost-efficient processes.

## Project chAnGE

It is becoming increasingly important to find ways to promote healthy ageing, which is defined as the process of developing and maintaining the functional ability that enables well-being in older age [14]. Project chAnGE (Clinical practice oriented cHange solutions towards Active aNd healthy aGEing) is a commitment from Upjohn (Division of Pfizer) and the European Innovation Partnership on Active and Healthy Ageing (EIP on AHA) initiative of the European Commission [15, 16]. One objective of Project chAnGE is to identify challenges to healthy and active ageing in people with NCDs in Europe. Project chAnGE is based on collaboration between private and public sectors, and such an integration is necessary to improve the care of NCDs. One of the priority areas that has been identified by Project chAnGE is the problem of fragmented care for older people with NCDs; many receive disjointed care from numerous specialists or via different levels of care. This increases the risk of inappropriate prescribing as many healthcare systems in Europe do not comprehensively implement electronic prescription systems to share information on prescriptions and treatment plans between care providers, pharmacists, and other health care professionals involved in the treatment of older persons with NCDs. Fragmented care and inappropriate prescribing can lead to low adherence, both in terms of drug adherence and adherence to care plans and non-pharmaceutical treatments. Low adherence is associated with multiple negative outcomes for patients in terms of disease progression, unplanned hospitalizations, and disability, and results in issues for national health services and society through increased costs for drugs that are dispensed but not taken, higher care needs, and rising unplanned hospitalization costs [17, 18]. Therefore, integrated care is essential for improving treatment adherence and improving outcomes for older patients with NCDs.

## Integrated care models

Although various definitions are available, integrated care generally refers to a coherent set of methods to align, connect, and increase collaboration between different administrative, organizational, service delivery and clinical levels in the care of a patient, to provide comprehensive and multidimensional treatment and care. Several approaches to improve the care process of chronic patients through integrated care have been proposed [14, 19], including the Chronic Care Model [20], the WHO Guidelines on Integrated Care for Older People (ICOPE) [19, 21], and the Joint Action CHRODIS Multimorbidity Care Model [22]. Such models usually consist of several individual components, for instance multidisciplinary comprehensive assessment, application of individualized care plans, integration between prescribers and pharmacists, medication reconciliation, use of case managers to coordinate care, and self-management support, among others. The current application of these components in clinical practice is varied, particularly for patients with multimorbidity [23].

## Healthy ageing in Europe: current barriers and solutions

In terms of the various components of integrated care Project chAnGE has identified several barriers, potential solutions, and mitigating actions to healthy ageing in Europe. It is important that we emphasize that there are large variations between European countries, but some common issues as well as coherent tools and approaches can be identified, as described below and in Table 1.

**Table 1:** Current gaps, possible effects and potential solutions for implementing and improving integrated care for the management of ageing-related non-communicable diseases (NCD)

Gaps and needs	Possible effects	Potential solutions
Underuse of case managers & care coordinators	Lack of care coordination, leading to fragmented healthcare Inappropriate polypharmacy such as over-prescribing	Widespread use of case managers for complex NCD patients
Lack of evidence-based guidelines for patients with multiple NCDs (multimorbidity)	Inadequate adaptation of single-disease-specific guidelines to complex patients Poor health outcomes for fragile, multimorbidity patients, including potential adverse drug interactions	Development and utilization of evidence-based guidelines for persons with multiple comorbid NCDs
Inadequate training for medical students and healthcare professionals on complex patients (multimorbidity, polypharmacy etc.)	Fragmented care and related negative health outcomes such as increased hospitalization, polypharmacy, adverse drug reactions etc.	Universal introduction of courses on complex NCD needs within basic medical training programs Family-physician and nurse practitioner training on integrated care
Focus on somatic health and single NCDs	Insufficient attention to the coexistence of both physical and psychiatric symptoms in persons with NCDs	More inter-sectorial care that combines social, psychological, and healthcare Extensive use of comprehensive geriatric assessments that measure patients’ somatic and psychiatric status
Underuse of healthcare-related ICT in in clinical practice	Difficulties in information sharing between different care levels and departments Lack of communication between prescribers and pharmacies leading to potential adverse drug reactions	Assignment more financial resources to both develop and support ICT use Development of better infrastructures to support use of healthcare ICTs ICT training for healthcare professionals
Heterogeneity of services and organization of NCD care across EU countries and lack of good quality evidence on effectiveness of integrated care interventions	Difficulties in applying universal integrated healthcare solutions across Europe	Evaluation integrated care models as a whole (rather than individual components) More European cross-country comparative research Development of methodological approaches for integrated care that can be adapted to different national health settings

*NCD* non-communicable diseases, *ICT* information and communication technology

### Case managers/care coordinators

One barrier is that case managers are not commonly used in some European settings. Moreover, in cases where case managers are utilized, other healthcare providers and patients sometimes do not recognize their role or utilize them appropriately. Most integrated care models highlight that it is essential to assign a case manager (sometimes referred to as a care coordinator), who is a health professional in charge of the integrated care of patients. Patient-centered care that focuses on shared decision-making should be enhanced. In a structured review of 18 papers [23] nurse case management was found to positively impact five health outcomes: objective clinical measurements, quality of life and functionality, patient satisfaction, adherence to treatment, and self-care and service use. It is, therefore, recommended that patients are provided with a care manager, who is responsible for coordinating their care and treatment in cases where healthcare is fragmented (both in terms of receiving care from separate specialists and across different levels of care). In situations where care managers are already available, they need to be better recognized and utilized by both patients and other healthcare providers.

### Disease-specific models and guidelines in populations with a high prevalence of multimorbidity

Another obstacle is that, despite the high prevalence of multimorbidity in the older population [13], there is currently a focus on disease-specific guidelines and care models that do not take comorbid conditions into account. It is therefore essential to continue developing evidence-based guidelines (e.g. Muth et al. [24, 25]) to design care pathways that are sensitive to the needs of multimorbid patients. These guidelines should also be developed in a simplified format to help patients and lay persons comprehend them, particularly the elements that require specific action from the patients themselves, for example lifestyle changes (nutrition, physical activity, etc.). Patients and their caregivers should be able to understand and navigate complex care pathways as well as recognize the potential interventions and healthcare options that may be relevant to them.

### Training and education

An additional hurdle is that training and education for medical students and healthcare professionals (especially in primary care) to address multimorbid patients, polypharmacy, chronicity, and complexity is lacking. To facilitate successful integrated care training is needed by healthcare professionals in many of the vital elements, including geriatric care [26, 27]. More information about the usefulness of training is expected from the ongoing “Improving Prescription in Primary Care Patients with Multimorbidity and Polypharmacy-Multi-PAP” project in Spain [28], which includes an intervention for patients with multimorbidity and polypharmacy consisting of family-physician training (on topics such as multimorbidity, appropriate prescribing, and shared decision making). This is one of the few studies that specifically investigates a training element for healthcare professionals, and thus the results will be invaluable for designing and implementing future integrated care models within Europe.

### Focus on somatic and mental health

Healthy ageing encompasses well-being both in terms of somatic and mental health. However, in current clinical practice in geriatric settings there is often insufficient attention on the coexistence of both physical and psychiatric symptoms in persons with NCDs. Thus, more inter-sectorial care is needed that combines social, psychological, and healthcare. Further, comprehensive geriatric assessment [12, 22, 29] should be a basic element of all integrated care models in the older population, as this allows a broad evaluation of multiple aspects of the patients physical, cognitive, and emotional well-being.

### Information and communication technology in healthcare

There is widespread consensus that Information and Communication Technology (ICT) is a valuable instrument for increasing healthy ageing and applying integrated care in clinical practice [15, 16]. However, it is a major barrier that ICT and eHealth tools are underused in European clinical settings either due to lack of resources, limited availability of well-designed healthcare technology, inadequate infrastructure to implement ICTs, or insufficient training. For example, Electronic Health Records need to be more widely used and they need to cover a broader range of variables, including information on symptoms and diagnoses, examinations, drug prescription, side effects etc. Importantly, these should be accessed and utilized by all healthcare providers involved in the patient’s care and linked nationally between care settings and pharmacies. Similarly, ICTs can be used to improve clinical decision support. An intervention to increase clinical decision support (consisting of patient adherence reports to healthcare providers plus email notifications to care managers) was effective in identifying medication adherence deficits and in increasing care manager responses to medication adherences issues [30]. New technologies can also help to support independence in multimorbid and frail patients and need to be specifically developed for these target groups.

### Evidence-based care

Finally, a major barrier is that there is currently a lack of good quality evidence that evaluates the effectiveness of integrated care interventions. It is likely that there are also geographical differences in care systems and availability of services, which need to be specifically studied. However, the current available evidence is promising. A systematic review of scientific papers investigating the success of chronic care models found that almost all reported either an improvement in clinical practice or health outcomes for patients with NCDs [31]. Another systematic review on the economic evidence suggested that integrated care programs have the potential to be cost-effective, achieving greater health benefits for patients while also being less expensive than usual care [32]. The reduction in costs is mainly due to a decrease in inpatient and outpatient admissions. However, it should be noted that much of the current evidence comes from clinical settings in the USA and there are limited well-designed trials in Europe, though results from studies such as the German-based PRIMUM trial [33] will soon help to provide a clearer picture of integrated care solutions within European settings.

## Further research

Moving forward, there is a need to investigate how integrated care models can be applied in different European health services, both in terms of countries and care settings (primary and community care, acute care, and specialized medical settings). Several trials are currently ongoing that may provide useful insight into how the various components of integrated care models can be applied in different European countries. For example, Joint Action CHRODIS+ is currently piloting an integrated care model for multimorbid patients in five European sites in three countries (Italy, Lithuania and, Spain) with the aim to develop an implementation model that can be adapted according to local and national needs [34]. In addition, findings from the Spanish Multi-PAP project described above are expected soon [28]. Further, Project chAnGE is supporting a number of projects in Europe to assess integrated care programs, for example for patients with non-malignant chronic pain in Italy and Portugal [16]. Results from pragmatic randomized control trials testing different aspects of integrated care are needed to implement it at population level. However, it is also important for research studies to move away from investigating how individual components impact patient outcomes and instead evaluate integrated care models as a whole. Further, it is essential to investigate how the results of integrated care models differ according to the characteristics and clinical complexity of the patient. For example, a recent trial from the UK, the “3D” trial [35] reported that a patient­centered care model with twice-yearly comprehensive multidisciplinary assessments did not improve patient outcomes in multimorbid patients. However, it was suggested that the lack of positive results may be due to the fact that they did not apply patient stratification techniques to identify the most demanding and complex-to-treat groups, who might benefit more from the care intervention [36]. Therefore, future research should focus on establishing whether integrated care is more successful if patients are stratified according to patterns of different diseases and symptoms or whether outcomes are more pronounced in patients with specific physical, cognitive, or socioeconomic characteristics. Finally, it is essential to consider the high heterogeneity of services and organization of care across EU countries. This limits the definition of standardized approaches to integrated care and calls for the identification of methodological approaches to integrated care rather than to the identification of one universal solution. The integration and comparison of data from different European health settings will aid this process.

## Conclusions

European health systems need to increase the use of integrated care to better manage patients with NCDs, which should hopefully help to improve treatment adherence and increase healthy ageing among the older population. The success of integrated care depends on the application of multiple, coordinated strategies rather than the individual components and, thus, healthcare providers should focus on identifying current fragmentations and delivering comprehensive care. Different level of the healthcare ecosystem should be addressed to make sure that a vertical integration of processes with common objectives are implemented. Moreover, the involvement of decision-makers is crucial to allow organizational and infrastructural changes that could facilitate the shift to more comprehensive approaches. Social, cultural, and educational barriers as well as limited resources are challenging the implementation of integrated care approaches in real-world practice. Scaling up health knowledge at the European level requires sharing integrated data [37]. A multidisciplinary, multi-institutional effort involving both public and private entities is necessary to implement new models that are both cost-effective and sustainable from a social and economic perspective.
